# Supplementary material for: Learning curve and surgical outcome of robotic assisted colorectal surgery with ERAS program
Source: Sci Rep. 2022 Nov 29;12:20566. doi: 10.1038/s41598-022-24665-w (PMC9709162; doi:10.1038/s41598-022-24665-w)
Supplement: Supplementary file 1 — Supplementary Information 1. [file 41598_2022_24665_MOESM1_ESM.zip › LSC Raw data-Ñ[▒K/080/H_ñw╝╨░O▒Kñσ.pdf]

正式摘要(電子簽章：00000192359615)

W55 / W55 ☐ 英文標題(注意:無英文姓名-請補病人基本檔!) ☐【讀取】憑證

臺中榮民總醫院

出院病歷摘要

轉入號： 轉出號：

[redacted]

(8)流水編號	
(9)入院日期	109/06/02 (2020) 直腸外科 W55 - 263
(10)轉科日期	
(11)出院日期	109/06/09 (2020) 住院天數計 7 日

診斷

- 入院 :Colon cancer, adenocarcinoma, moderately differentiated 55cm FAV, s/p loop distal T-colostomy on 20200514
- 出院 :1.Colon cancer, adenocarcinoma, moderately differentiated 55cm FAV, s/p loop distal T-colostomy on 20200514, s/p laparoscopic Anterior resection 20200603, pT3N0(0/18) Mo, stage IIA, G2 MD, AnI-, PI:-, Tudp:-, Pefr:-, CRM>10mm, ExNd:-.
  2.Gall stone, s/p Laparoscopic Cholecystectomy on 20200603

主訴

- nausea vomiting for 1 m. s/p loop T colostomy.

病史

- This 71-year-man has history of
  1.Colon cancer, adenocarcinoma, moderately differentiated 55cm FAV, s/p loop distal T-colostomy on 20200514
  2.Gall stone
  3.bilateral hernia
  According to patient,he sufferd from abdomen distention since 20200508. Constipation for 4 days.He ever came to 南投醫院ER on 2020/05/11.In where,abdomen CT showed ileus and S colon lesion, arrange admitted to him. But he refuse, AAD and transfer to our ER .He denied fever,SOB, DOE, cold sweating,diarrhea, tarry or bloody stool.TOCC(-). At our ER, initial vital signs revealed BP: 159/90mmHg , PR:80bpm, RR:18 cpm , BT: 35.9 'C.Physical exam revealed clear breath sound , no rales,abdomen distention,soft, normactive bowel sound, tenderness over A4,6,7 and less rebounding pain.Lab data revealed WBC 9750,NEUT80.9%,k3.4meq/l,CRP 0.637mg/dl.The CXR revealed no obvious pneumonia patch. KUB showed intestinal obstruction.Refuse NG sump insertion.S colonscope showed S-colon ulcers, s/p Bx at 30cm from AV.Under tentative diagnosis of intestinal obstruction and r/o colon mass,NPO and iv hydration,cefazolin and metronidazole use.He was admitted for further treatment. After admission, loop distal T-colostomy on 20200514 was done and discharge on 5/22. Pathology showed adenocarcinoma, moderately differentiated. Therefore, this time he was admitted for laparoscopic anterior resection with anastomosis and laparoscopic Cholecystectomy on 20200603

體檢發現

- CEA: 12(2020/5)

  2020/5 PET: S colon ca.(Gr4). para stoma lesion(Gr1)

  - General appearance: A well-nourished person, with acute ill looking. Consciousness: Alert Pupil size: 3.5 mm Light reflex:
    - Normal light reflex

    Vital signs:

date	體溫(BT)	脈搏(P)	呼吸(R)	血壓(BP)	身高(BH)	體重(BW)	BMI
20200602	35.5℃	89次/分	18次/分	112/84mmHg	165.1cm	62kg	22.7
  - Skin
    - Normal skin turgor , No jaundice , No cyanosis , No rash , No wound / ulcer / scar
  - Lymphadenopathy
    - No lymphadenopathy

-
  - HEENT
    - Head: No shape deformity , No hair change , Normal lip
    - Eyes: ＊Conjunctiva: Normal , ＊Sclera: Not icteric , ＊Cornea: Clear , ＊ Eyelids: Normal
    - Ears: No deformity
    - Nose: Normal appearance
    - Mouth and throat: No oral mucosa change , No throat injection , No tonsil enlargement
  - Neck
    - No thyroid enlargement , Neck: Jugular vein
  - Chest
    - Inspection: Normal and symmetric respiratory movement , No deformity , Normal breast
    - Palpation: No palpable mass , No tender point
    - Auscultation: No rales , No rhonchi , No wheezing , Chest / Auscultation / Stridor No stridor , Not perform , No friction rub
  - Heart
    - Inspection: No visible apical impulse
    - Palpation: Not palpable PMI , No thrill , No heave
    - Auscultation: Regular heart beats , No S3 , No S4 , No murmur
  - Abdomen
    - Inspection: No distension , No striae , stomy
    - Auscultation: Normal bowel sound ,
    - Palpation: No hepatomegaly , No tenderness , No rebounding pain , No palpable mass , No splenomegaly
    - Percussion: No shifting dullness
  - Genito-anus patient refuse
  - Back and spine
    - No deformity , No knocking pain
  - Extremities
    - No deformity , No clubbing finger , No pitting edema , Normal joint , Normal peripheral pulse
  - Neurological examination
    - No mental status change

手術日期及方法

- 手術日期及方法20200603 Laparoscopic Cholecystectomy
  20200603 Laparoscopic Anterior resection and anastomosis - malignant

住院治療經過

- During admission, laparoscopic anterior resection with anastomosis and cholecystectomy was done on 20200603. After surgery, we removed NG on 6/4 and removed foley on 6/5. Due to patient's penrose drainage amount decreased and we removed penrose on 6/8 and removed stitches on 6/9. Because patient's condition became stable, patient would discharge and f/u at OPD.

合併症

一般檢查

- 累積報告 :

DATE	TIME	ALB	TP	BIL.T	BIL.D	ALKP	AST	ALT	LDH	CHOL	TG	HDL-C	LDL	UA	NH3	AMY
1090511	2022	4.6		1.1		81		43	218							
1090514	1701	3.6		0.8				16								
1090518	0737	2.8		0.49				4								
1090521	0925	3.3		0.30				10								
1090525	1439	3.8		0.61	0.19	80	55	70	217							
1090604	0929	3.3					45	42								
1090608	0916	2.9	5.0	0.37	0.12	57	16	9	158							

DATE	TIME	WBC	RBC	HGB	HCT	MCV	PLT	BLST	NEUT	BAND	SEG	LYM	MON	EOS	BAS
1090511	2021	9750		16.9			213		80.9			12.1	6.4	0.2	0.4
1090514	1700	5490		16.6	50.6		197		76.9			12.6	9.8	0.2	0.5

1090515	1655	4280	4.92	15.0			166		57.5			24.5	17.3	0.2	0.5
1090518	0736	7640	4.68	14.0			201		78.1			9.2	10.2	2.1	0.4
1090521	0859	9320	5.25	15.5			262		72.0			16.6	7.9	3.0	0.5
1090525	1439	9760	5.81	17.3	54.2	93.3	349		76.2			16.8	4.8	1.4	0.8
1090604	0927	13010	4.60	13.8	42.3	92.0	208		83.6			8.0	8.2	0.1	0.1

DATE	TIME	NA	K	CL	CA	BUN	CREAT	Mg	P	CRP	ESR	PCT	LIPASE	LACTATE
1090511	2022	143	3.4	107	9.0	12	1.07			0.637				31.1
1090512	0602	142	3.2											12.9
1090514	1701	147	3.4		7.9	11	1.09							16.5
1090515	1651													15.4
1090515	1655									9.261				
1090518	0737	138	2.8	109	7.2	10	0.91							
1090521	0925	139	3.9	107	8.3	14	0.91							
1090525	1439	141	4.6		9.0	17	1.12							
1090604	0929	141	3.7	107	8.1	9	0.89							
1090608	0916	142	3.6	110	8.0	9	0.85							

DATE	TIME	GLUCOSE	PAB	TRF	ZN	CU	KETONE	CK	CKMB	PT-P	PT-C	APTT-P	APTT-C	TRO-T
1090511	2022	120								11.4	10.3	28.8		
1090525	1439	191								11.4	10.3	27.9		

DATE	TIME	SP-GR	PH	PROT	GLUC	KETON	BILI	UROB	OB	NITR	LEU	RBC	WBC	EPCEL
1090515	1918	1.020	6.5	1+	250.0	Trace	1+	1.0	Negative	Positive	Trace	0-2	0-2	0-1

DATE	TIME	PH	PCO2	PO2	SO2	BEB	HCO3	TCO2	HGB	FIO2
1090511	2022	7.254	55.6	17.3	17.9	-4.2	24.0	25.7		

特殊檢查

病理報告

其它

出院時情況

- 3.改門診治療

出院指示

藥名	劑量	劑量單位	用法	頻次	數量	數量單位	現況	天數	單位
OCAS Harnalidge 0.4 mg	1	TAB	PO	QDAC	7	TAB	TH	7	天
Lactam tab 500mg	1	TAB	PO	Q8HPRN	21	TAB	TH	7	天

出院計劃

主治醫師 2222H 林俊余 / 住院醫師 9922K 洪宇漢

正式摘要**(電子簽章：000000192359615)**

W55 / W55

doctorRank:B
